# Exploring the Role of Turbinate Reduction in Alleviating Attention-Deficit/Hyperactivity Disorder (ADHD) Symptoms in Pediatric Patients

**DOI:** 10.7759/cureus.88807

**Published:** 2025-07-26

**Authors:** Ahmed H Ali, German P Digoy

**Affiliations:** 1 Pulmonary, University of Oklahoma Health Sciences Center, Oklahoma City, USA; 2 Pediatric Otolaryngology, Oklahoma State University Center for Health Sciences, Tulsa, USA

**Keywords:** attention deficit hyperactivity disorder (adhd), behavioral improvement, bipolar cautery, inferior turbinate reduction, nasal airway obstruction, nasal surgery, pediatric nasal obstruction, retrospective cohort study, sleep-disordered breathing

## Abstract

Background

Sleep-disordered breathing (SDB) and attention-deficit/hyperactivity disorder (ADHD) symptoms frequently co-occur in children and may be exacerbated by nasal obstruction. While adenotonsillar hypertrophy has been extensively studied, the role of inferior turbinate hypertrophy remains underrecognized.

Objective

To evaluate the effects of inferior turbinate reduction (ITR) on nasal breathing, SDB symptoms, and ADHD-type behaviors in pediatric patients.

Methods

We performed a retrospective cohort study of children undergoing ITR between December 2020 and May 2023. Patient selection included all children aged 3-17 years who underwent ITR via bipolar cautery, either as an isolated procedure or combined with adenotonsillectomy, septoplasty, or functional endoscopic sinus surgery. Twelve-month parent-reported outcomes were collected through chart reviews and supplemental telephone interviews when needed. Primary outcomes included subjective improvement in nasal breathing, resolution of SDB symptoms (snoring, witnessed apneas, restless sleep), and parent-reported changes in ADHD-type behaviors (hyperactivity, inattention, impulsivity). Statistical analyses included McNemar tests, χ²/Fisher's exact tests, and multivariable logistic regression.

Results

Among 326 children (mean age 9.0±4.1 years; 212/326 [65%] male), 106/326 (32.5%) had baseline ADHD-type symptoms. Concurrent procedures included adenotonsillectomy (198/326 [60.7%]), septoplasty (45/326 [13.8%]), and functional endoscopic sinus surgery (24/326 [7.4%]). In the ADHD subgroup, 95/106 (89.6%) reported improved nasal breathing, 89/106 (84.0%) had resolution of SDB symptoms, and 51/106 (48.1%) reported improvement in ADHD symptoms. Improvement in ADHD symptoms was significantly associated with nasal breathing improvement (χ²=11.38, p<0.001), although no independent predictors were identified on logistic regression. In the ITR-only subgroup (59/326 [18.1%]; 21/59 with ADHD), 11/21 (52.4%) reported ADHD improvement, with no significant difference compared to patients undergoing concurrent procedures (p=0.66).

Conclusion

ITR is associated with significant improvements in nasal obstruction, SDB, and ADHD-related behaviors. However, the retrospective design, lack of control group, and absence of validated assessment tools limit causal inference. Prospective controlled studies utilizing validated instruments are essential to establish causality.

## Introduction

Sleep-disordered breathing (SDB) and attention-deficit/hyperactivity disorder (ADHD) represent two of the most prevalent pediatric conditions, affecting 1-5% and 5-7% of children, respectively [1-3]. Their frequent co-occurrence has prompted investigation into potential shared pathophysiological mechanisms. Nasal obstruction, a modifiable factor contributing to SDB, has emerged as a potential link between these conditions.

The relationship between upper airway obstruction and neurobehavioral symptoms is supported by multiple lines of evidence. First, chronic nasal obstruction leads to obligate mouth breathing, which disrupts normal sleep architecture through increased upper airway resistance and fragmented sleep [4,5]. Second, the resulting intermittent hypoxia and sleep fragmentation affect prefrontal cortex function - the brain region responsible for executive function, attention regulation, and impulse control [6,7]. Third, inflammatory mediators released during chronic upper airway obstruction may directly impact neurodevelopment and behavior [8]. While adenotonsillar hypertrophy has been extensively studied as a cause of pediatric SDB, with demonstrated behavioral improvements following adenotonsillectomy [9-11]. The role of inferior turbinate hypertrophy remains underrecognized. We therefore hypothesize that relieving nasal obstruction with inferior turbinate reduction improves airflow, which diminishes sleep‑fragmentation‑driven SDB and, in turn, attenuates ADHD‑type behaviors. The inferior turbinates constitute the primary nasal resistance site, accounting for up to 50% of total nasal airway resistance [12]. Chronic turbinate hypertrophy, often resulting from allergic rhinitis or chronic rhinosinusitis, can significantly impair nasal breathing even in the absence of adenotonsillar pathology.

Inferior turbinate reduction (ITR) using bipolar cautery provides a minimally invasive approach to address turbinate hypertrophy. The procedure involves submucosal cauterization that induces controlled tissue reduction while preserving mucosal function. Despite its widespread use in pediatric otolaryngology, data on behavioral outcomes following ITR remain limited. This study examines the impact of inferior turbinate reduction on nasal breathing, SDB symptoms, and ADHD-type behaviors in a large retrospective cohort, with particular attention to the potential mechanisms linking nasal obstruction relief to behavioral improvement.

## Materials and methods

Study design and population

We conducted a retrospective review of medical records from a tertiary pediatric otolaryngology practice from December 2020 to May 2023. The study protocol received institutional review board approval.

Patients were included if they were aged 3-17 years at the time of surgery, underwent inferior turbinate reduction via bipolar cautery, had a minimum 12-month follow-up available, and parent/guardian consent was obtained for participation. All patients included in the study had documented failure of comprehensive medical therapy prior to surgical intervention. Standard medical management typically included intranasal corticosteroids (e.g., fluticasone), intranasal antihistamines (e.g., azelastine), oral antihistamines, leukotriene receptor antagonists (e.g., montelukast), allergy testing and immunotherapy when clinically indicated. Insurance authorization for Inferior turbinate reduction required documentation of failed medical therapy. Exclusion criteria comprised known neurodevelopmental disorders such as autism spectrum disorder or intellectual disability, chromosomal abnormalities including Down syndrome or 22q11.2 deletion syndrome, previous nasal surgery, incomplete follow-up data or inability to contact family, and declined consent for study participation.

Surgical technique

All procedures were performed under general anesthesia by fellowship-trained pediatric otolaryngologists. ITR was accomplished using bipolar cautery applied submucosally along the inferior turbinate length, with care taken to preserve the medial mucosal surface. Power settings ranged from 15-25 watts based on patient age and turbinate size. When performed, concurrent procedures followed standard techniques. Adenotonsillectomy involved adenoidectomy via suction cautery and tonsillectomy using monopolar cautery or coblation. Septoplasty utilized a conservative cartilage-sparing technique for symptomatic septal deviation. Functional Endoscopic Sinus Surgery (FESS) consisted of limited maxillary antrostomy and anterior ethmoidectomy for chronic rhinosinusitis.

Data collection

Medical records were systematically reviewed for comprehensive patient information. Baseline demographics included age at surgery and gender. Clinical characteristics encompassed the presence of ADHD-type symptoms defined as parent-reported hyperactivity, inattention, or impulsivity documented in preoperative notes, ADHD diagnosis and current medications, comorbid conditions including allergic rhinitis (with documentation of allergy testing results and immunotherapy status when available), asthma, and chronic rhinosinusitis, as well as previous medical treatments (specific medications tried and duration) and surgical interventions.

Surgical details captured included the primary procedure designation of ITR alone versus concurrent procedures, specific concurrent procedures performed, and intraoperative findings including turbinate size, septal deviation degree, and sinus disease extent. Outcome measures assessed at 12-month follow-up comprised nasal breathing improvement defined as parent-reported change from baseline categorized as improved, unchanged, or worse, SDB symptom resolution evaluated as a composite of snoring cessation, absence of witnessed apneas, and normalized sleep patterns, and ADHD-type behavior change assessed through parent-reported global assessment of hyperactivity, attention, and impulsivity categorized as improved, unchanged, or worse. When 12-month clinic follow-up data were incomplete, structured telephone interviews were conducted by trained research staff using a standardized script to obtain missing outcome data. The complete telephone script is provided in Supplemental Appendix A.

Statistical analysis

Statistical analyses were performed using SPSS version 30 (IBM Corporation, Armonk, NY, USA). Descriptive statistics included means ± standard deviations for continuous variables and frequencies with percentages for categorical variables. Within the baseline ADHD subgroup, paired pre- and post-surgery changes were evaluated using McNemar's test for dichotomous outcomes. Association between symptom improvements was assessed using χ² tests or Fisher's exact test for cell counts less than 5. Multivariable binary logistic regression assessed predictors of ADHD improvement, incorporating nasal breathing improvement (yes/no), SDB resolution (yes/no), sex (male/female), age at surgery (continuous), and concurrent procedures (yes/no). Model performance was evaluated using the Hosmer-Lemeshow goodness-of-fit test, Nagelkerke R², and 95% confidence intervals. Statistical significance was set at p<0.05.

## Results

Patient characteristics

**Figure 1:**
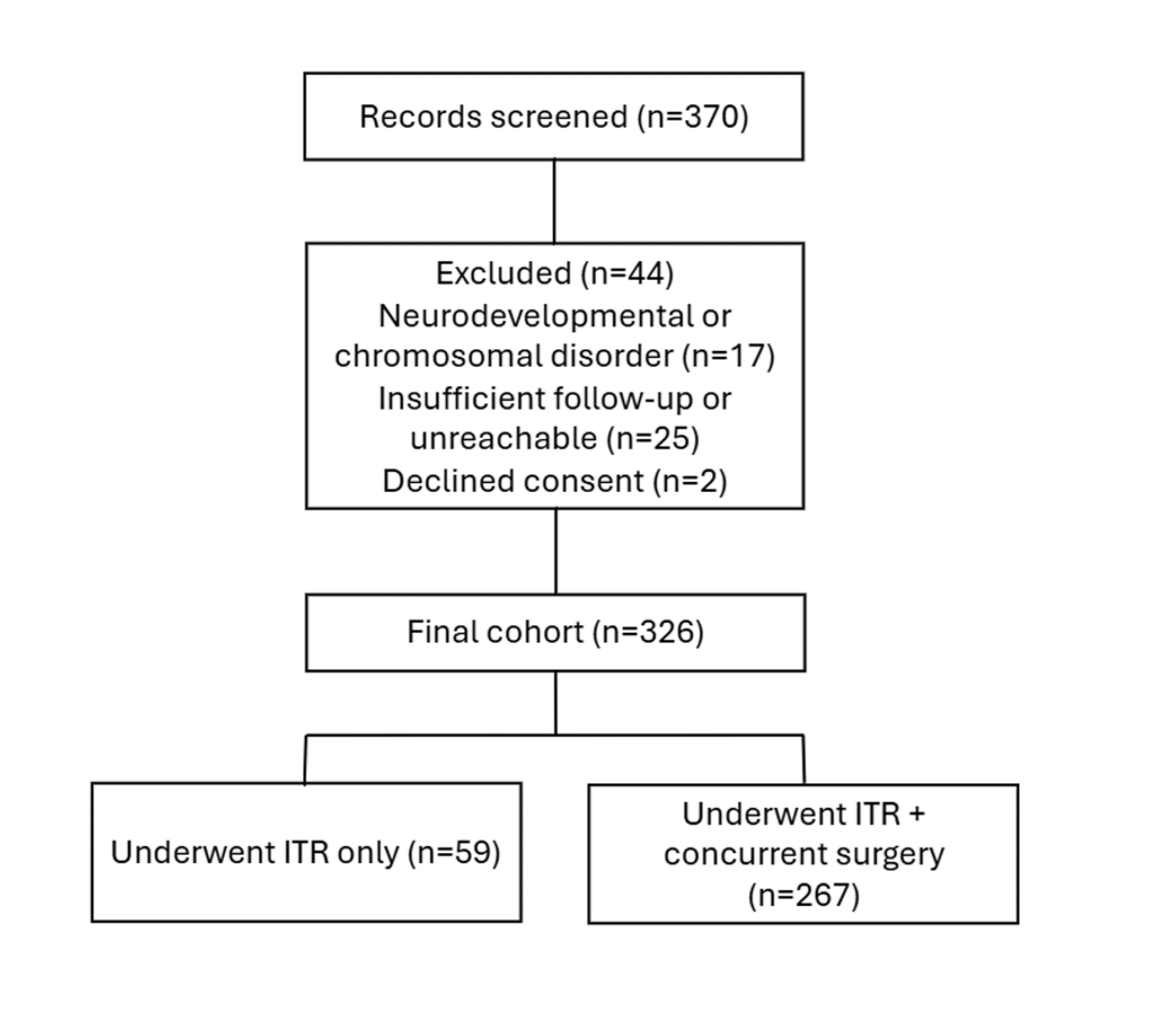
Flow diagram of patient screening and enrollment A total of 370 patients were screened, of whom 44 were excluded for neurodevelopmental/chromosomal disorders (n=17), insufficient follow-up or inability to contact (n=25), or declined consent (n=2), resulting in a final cohort of 326 patients who underwent inferior turbinate reduction (ITR), with or without concurrent procedures.

Of 370 patients screened, 44 were excluded (17 for neurodevelopmental/chromosomal disorders, 25 for incomplete follow-up or unreachable, and two who declined consent), resulting in a final cohort of 326 patients (Figure 1). The cohort included 326 children with a mean age of 9.03 ± 4.09 years (range 3-17 years). Males comprised 65.0% (n=212) of the sample. The broad age range reflected varying presentations: younger children (3-6 years) typically presented with adenotonsillar hypertrophy and turbinate enlargement, while older children (12-17 years) more commonly had isolated turbinate hypertrophy from allergic rhinitis.

Surgical procedures

ITR was performed as an isolated procedure in 59 (18.1%) patients. The majority (267, 81.9%) underwent concurrent procedures, including adenotonsillectomy in 198 (60.7%), septoplasty in 45 (13.8%), and functional endoscopic sinus surgery in 24 (7.4%). The high rate of concurrent adenotonsillectomy reflects the common co-occurrence of adenotonsillar and turbinate hypertrophy in pediatric nasal obstruction. Septoplasty was reserved for children >12 years with completed facial growth and symptomatic deviation. FESS was performed for medically refractory chronic rhinosinusitis with polyps or significant mucosal disease.

Primary outcomes

In the overall cohort (N=326), nasal breathing improvement was achieved in 301/326 (92.3%) patients, SDB resolution occurred in 182/209 (87.1%) among those with baseline SDB, and ADHD behavior improvement was observed in 51/106 (48.1%) among those with baseline ADHD symptoms. For the baseline ADHD subgroup analysis (n=106), Table 1 presents detailed symptom trajectories. All children with baseline ADHD symptoms had documented nasal obstruction preoperatively. Following surgery, 95/106 (89.6%) reported improved nasal breathing, 89/95 (93.7%) with baseline SDB achieved symptom resolution, and 51/106 (48.1%) demonstrated ADHD behavior improvement.

**Table 1:** Symptom Trajectories in the Baseline-ADHD Subgroup (n=106) * P-values calculated using McNemar's test for paired pre- vs. post-operative comparisons; all comparisons statistically significant at p < 0.001

Symptom Domain	Pre-op Yes n/N (%)	Post-op Yes n/N (%)	Paired P*
Nasal Obstruction	106/106 (100)	11/106 (10.4)	< 0.001
Sleep-disordered breathing	95/106 (89.6)	6/106 (5.7)	< 0.001
ADHD-type behavior	106/106 (100)	55/106 (51.9)	< 0.001

The association between nasal breathing improvement and ADHD improvement was statistically significant in univariate analysis (χ²=11.38, p<0.001). Among children with improved nasal breathing, 50.5% (48/95) reported ADHD improvement, compared to 27.3% (3/11) without nasal improvement. However, logistic regression modeling (Table 2) revealed no independent predictors of ADHD improvement. Despite the strong univariate association, nasal breathing improvement did not reach significance (OR 0.29, 95% CI 0.03-2.73, p=0.28). The model demonstrated poor fit (Nagelkerke R²=0.065), suggesting unmeasured confounders or complex interactions not captured by our variables.

**Table 2:** Multivariable Logistic Regression Predicting ADHD-symptom Improvement (n=106) Model fit statistics for the logistic regression analysis: −2 Log Likelihood = 141.6; Nagelkerke R² = 0.065, indicating modest explanatory power; Hosmer‑Lemeshow goodness-of-fit test χ² = 9.64 (df = 2, p = 0.008), suggesting lack of model fit.

Predictor	Odds Ratio (OR)	95% CI	P
Nasal-breathing improvement	0.29	0.03 - 2.73	0.28
SDB resolution	6.23	0.7-55.83	0.10
Male Gender	0.64	0.28 - 1.50	0.31
Age at surgery	1.04	0.94-1.15	0.44
Concurrent procedures	0.89	0.33-2.40	0.82

Among 59 children undergoing isolated ITR, 21 (35.6%) had baseline ADHD symptoms. In this ITR-only subgroup, ADHD improvement occurred in 11/21 (52.4%) with McNemar exact p=0.001, SDB resolution was achieved in 15/15 (100%) with baseline SDB, with McNemar exact p<0.001, and nasal breathing improvement was reported in 19/21 (90.5%). Comparison of ADHD improvement rates between ITR-only (52.4%) and concurrent surgery groups (47.1%) showed no significant difference (χ²=0.19, p=0.66), suggesting that behavioral improvements were primarily related to nasal airway enhancement rather than adenotonsillar removal.

## Discussion

This study represents one of the largest cohorts examining behavioral outcomes following inferior turbinate reduction in children. Our findings demonstrate high rates of nasal breathing improvement (89.6%) and SDB resolution (93.7%) following ITR, with nearly half of the children with baseline ADHD-type behaviors showing improvement at 12-month follow-up.

The observed behavioral improvements following ITR likely reflect multiple interconnected mechanisms that can be understood through a cascade of physiological effects. Nasal obstruction increases upper airway resistance, leading to increased respiratory effort, frequent arousals, and sleep fragmentation. ITR reduces nasal resistance, facilitating nasal breathing and improving sleep continuity [12,13]. This improved sleep continuity enhances sleep architecture, particularly increasing slow-wave and rapid eye movement (REM) sleep, which directly benefits attention and executive function [14]. For children receiving ADHD medications, this improved sleep quality may enhance medication effectiveness by optimizing baseline arousal and attention states. Chronic nasal obstruction can also cause intermittent hypoxia, particularly during sleep. Even mild intermittent hypoxia affects prefrontal cortex function - the brain region critical for attention regulation and impulse control [15]. By improving nasal patency, ITR may enhance cerebral oxygenation and prefrontal function. Additionally, SDB triggers sympathetic nervous system activation, elevating catecholamine levels that persist into waking hours. This chronic sympathetic activation mimics and potentially exacerbates ADHD symptoms, and resolving SDB through improved nasal breathing may normalize autonomic function [16,17]. Finally, chronic upper airway obstruction promotes local and systemic inflammation through hypoxia-induced cytokine release. These inflammatory mediators can cross the blood-brain barrier and affect neurotransmitter systems implicated in ADHD, suggesting that ITR may reduce this inflammatory burden [18-21].

The 48.1% ADHD improvement rate suggests that nasal obstruction evaluation should be routine in children with ADHD symptoms. While not all children benefit behaviorally, the high success rate for nasal breathing and SDB symptoms, combined with low procedural morbidity, supports considering ITR in appropriately selected patients. The similar improvement rates between ITR-only and concurrent procedure groups suggest that turbinate reduction alone may suffice for some children, potentially avoiding more invasive procedures. However, individual anatomy and obstruction sites must guide surgical planning.

Several important limitations must be acknowledged in interpreting these findings. The absence of a non-surgical control group precludes definitive causal inference, as observed improvements could reflect placebo effects, natural symptom fluctuation, or regression to the mean. A randomized controlled trial comparing ITR to medical management would address this limitation. Additionally, ADHD-type behaviors were identified through chart review and parent report rather than standardized instruments like the Vanderbilt ADHD Rating Scale or Conners Rating Scales. This subjective assessment may introduce bias and limit comparison with other studies. The lack of standardized diagnostic criteria and psychological testing limits our ability to distinguish true ADHD from ADHD-like symptoms secondary to sleep disruption. We also could not account for all potential confounding factors including ADHD medication changes during follow-up, behavioral interventions or therapy, comorbid conditions such as anxiety or learning disabilities, environmental factors like school changes or family stressors, and allergic rhinitis treatment optimization. The 3-17-year age range encompasses different developmental stages with varying ADHD presentations and surgical indications, though age-stratified analyses were limited by sample size. Furthermore, we relied on subjective parent reports rather than objective measures such as polysomnography to document SDB resolution, acoustic rhinometry or rhinomanometry for nasal patency, neuropsychological testing for attention and executive function, or teacher ratings for behavioral changes, therapy, standardized assessment and documentation of allergic rhinitis severity and treatment response. Results from a tertiary referral center may not generalize to community settings with different patient populations and surgical expertise. Moreover, because children were selected for surgery only after failing medical therapy, our cohort likely skews toward more severe cases, which may inflate treatment‐response estimates and limit generalizability. Finally, the 25 excluded patients lost to follow-up may have had different outcomes, potentially biasing results if loss to follow-up was related to treatment success or failure.

Several non‑surgical factors could also have contributed to the observed behavioral gains. Natural developmental maturation, optimization of allergic‑rhinitis management during follow‑up, and psychosocial changes at home or school may independently improve attention and sleep. These potential confounders underscore the need for randomized controlled designs that capture medication adjustments, allergy‑therapy response, and environmental variables.

This study provides hypothesis-generating data supporting prospective investigation. Future studies should employ randomized controlled designs comparing ITR to medical management, utilize validated ADHD assessment tools pre- and post-operatively, include objective measures such as polysomnography, rhinomanometry, and neuropsychological testing, stratify by age, ADHD subtype, and baseline severity, subgroup analyses based on allergic rhinitis presence, severity, and treatment response, control for medication changes and behavioral interventions, extend follow-up beyond 12 months to assess durability, and investigate biomarkers linking nasal obstruction to behavioral symptoms.

## Conclusions

Inferior turbinate reduction is associated with substantial improvements in nasal obstruction, near-universal SDB resolution, and clinically meaningful behavioral improvements in approximately half of children with ADHD-type symptoms. While these findings are encouraging, the retrospective design and methodological limitations preclude establishing causality. Prospective controlled trials using validated assessment instruments are essential to confirm these associations and establish ITR's role in the multidisciplinary management of children with comorbid nasal obstruction and ADHD symptoms. Until such evidence is available, ITR should be considered primarily for its established benefits on nasal breathing and sleep, with behavioral improvement viewed as a potential secondary benefit requiring further validation.

## Appendices

Appendix A

1. Greeting / Identity Check

   “Hello, may I speak with {Parent Name}?  This is {Your Name} calling on behalf of Dr. Digoy’s office about your child {Child Name}’s nasal surgery last year. Is this a good time to talk? This will take about 5 minutes.”

   → If parent declines: “No problem, when would be a convenient time for me to call back?”

2. Verbal Consent

   “Great, thank you. I’d like to ask a few routine follow‑up questions about your child’s breathing, sleep, and behavior. Your responses will be kept confidential and used for quality improvement research. May I proceed?”

   ► If no → End call, note refusal.

3. Nasal Breathing

   “Since the surgery, would you say your child’s nasal breathing is:

     a) Improved

     b) Unchanged

     c) Worse”

4. Snoring

   “Before surgery your child snored during sleep. Do they still snore? (Yes/No)

     If Yes: Is it

      • Less frequent

      • About the same

      • More frequent?”

5. Witnessed Apneas / Gasping

   “Have you observed pauses in breathing or gasping while your child sleeps? (Yes/No)

     If Yes: Are these

      • Less frequent

      • About the same

      • More frequent?”

6. Restless Sleep

   “Does your child still toss and turn or seem restless at night? (Yes/No)

     If Yes: Less / Same / More?”

7. Hyperactivity

   “In terms of daytime hyperactivity, have you noticed:

     a) Improvement

     b) No change

     c) Worsening?”

8. Attention / Focus

   “How about your child’s ability to pay attention or stay focused?

     a) Improved

     b) Unchanged

     c) Worse”

9. Impulsivity

   “And impulsive behaviors such as acting before thinking-improved, unchanged, or worse?”

10. Overall Sleep Quality

    “Overall, would you say your child’s quality of sleep has:

      a) Improved

      b) Remained the same

      c) Worsened

      d) Not sure?”

11. ADHD Medication Changes

    “Since the surgery, has there been any change in your child’s ADHD medications?

      • Stopped medication

      • Dose decreased

      • Dose increased

      • New medication started

      • No changes / not on medication”

12. Post‑operative Complications

    “Has your child experienced any nasal problems such as bleeding, crusting, or infection since surgery? (Yes/No)  If Yes, please describe.”

13. Other Health Events

    “Have there been any other surgeries or major health events for your child in the past year? (Yes/No)  If Yes, please specify.”

14. Open Comment

    “Is there anything else you’d like to share about your child’s progress?”

15. Closing

    “Thank you very much for your time and feedback. If you have any concerns, please feel free to call us at (405) 757-8677.  Have a great day.”

## References

[1] Marcus CL, Brooks LJ, Draper KA (2012). Diagnosis and management of childhood obstructive sleep apnea syndrome. Pediatrics.

[2] Thomas R, Sanders S, Doust J, Beller E, Glasziou P (2015). Prevalence of attention-deficit/hyperactivity disorder: a systematic review and meta-analysis. Pediatrics.

[3] Sedky K, Bennett DS, Carvalho KS (2014). Attention deficit hyperactivity disorder and sleep disordered breathing in pediatric populations: a meta-analysis. Sleep Med Rev.

[4] Guilleminault C, Khramtsov A (2001). Upper airway resistance syndrome in children: a clinical review. Semin Pediatr Neurol.

[5] Chervin RD, Archbold KH, Dillon JE, Panahi P, Pituch KJ, Dahl RE, Guilleminault C (2002). Inattention, hyperactivity, and symptoms of sleep-disordered breathing. Pediatrics.

[6] Beebe DW, Gozal D (2002). Obstructive sleep apnea and the prefrontal cortex: towards a comprehensive model linking nocturnal upper airway obstruction to daytime cognitive and behavioral deficits. J Sleep Res.

[7] Tamanyan K, Weichard A, Biggs SN, Davey MJ, Nixon GM, Walter LM, Horne RS (2019). The impact of central and obstructive respiratory events on cerebral oxygenation in children with sleep disordered breathing. Sleep.

[8] Huang YS, Guilleminault C, Hwang FM, Cheng C, Lin CH, Li HY, Lee LA (2016). Inflammatory cytokines in pediatric obstructive sleep apnea. Medicine (Baltimore).

[9] Mitchell RB, Kelly J (2007). Behavioral changes in children with mild sleep-disordered breathing or obstructive sleep apnea after adenotonsillectomy. Laryngoscope.

[10] Marcus CL, Moore RH, Rosen CL (2013). A randomized trial of adenotonsillectomy for childhood sleep apnea. N Engl J Med.

[11] Isaiah A, Pereira KD, Das G (2019). Polysomnography and treatment-related outcomes of childhood sleep apnea. Pediatrics.

[12] El-Anwar MW, Hamed AA, Abdulmonaem G, Elnashar I, Elfiki IM (2017). Computed tomography measurement of inferior turbinate in asymptomatic adult. Int Arch Otorhinolaryngol.

[13] Farmer SE, Eccles R (2006). Chronic inferior turbinate enlargement and the implications for surgical intervention. Rhinology.

[14] Scullin MK, Gao C (2018). Dynamic contributions of slow wave sleep and REM sleep to cognitive longevity. Curr Sleep Med Rep.

[15] Bass JL, Corwin M, Gozal D (2004). The effect of chronic or intermittent hypoxia on cognition in childhood: a review of the evidence. Pediatrics.

[16] Hakim F, Gozal D, Kheirandish-Gozal L (2012). Sympathetic and catecholaminergic alterations in sleep apnea with particular emphasis on children. Front Neurol.

[17] Vardhan V, Shanmuganandan K (2012). Hypertension and catecholamine levels in sleep apnoea. Med J Armed Forces India.

[18] Tam CS, Wong M, McBain R, Bailey S, Waters KA (2006). Inflammatory measures in children with obstructive sleep apnoea. J Paediatr Child Health.

[19] Anand D, Colpo GD, Zeni G, Zeni CP, Teixeira AL (2017). Attention-deficit/hyperactivity disorder and inflammation: what does current knowledge tell us? A systematic review. Front Psychiatry.

[20] Song Y, Lu M, Yuan H, Chen T, Han X (2020). Mast cell-mediated neuroinflammation may have a role in attention deficit hyperactivity disorder (Review). Exp Ther Med.

[21] Steinke JW, Woodard CR, Borish L (2008). Role of hypoxia in inflammatory upper airway disease. Curr Opin Allergy Clin Immunol.

